# Determination of oxidative stress and activities of antioxidant enzymes in guinea pigs treated with haloperidol

**DOI:** 10.3892/etm.2012.822

**Published:** 2012-11-20

**Authors:** JAROMIR GUMULEC, MARTINA RAUDENSKA, MARIAN HLAVNA, TIBOR STRACINA, MARKETA SZTALMACHOVA, VERONIKA TANHAUSEROVA, LUKAS PACAL, BRANISLAV RUTTKAY-NEDECKY, JIRI SOCHOR, ONDREJ ZITKA, PETR BABULA, VOJTECH ADAM, RENE KIZEK, MARIE NOVAKOVA, MICHAL MASARIK

**Affiliations:** 1 Department of Pathological Physiology, Faculty of Medicine; Brno University of Technology, Brno, Czech Republic; 2 Department of Physiology, Faculty of Medicine, Masaryk University; Brno, Czech Republic; 3 Department of Chemistry and Biochemistry, Faculty of Agronomy, Mendel University in Brno; Brno, Czech Republic; 4 Central European Institute of Technology, Brno University of Technology; Brno, Czech Republic; 5 Department of Veterinary Ecology and Environmental Protection, Faculty of Veterinary Hygiene and Ecology, University of Veterinary and Pharmaceutical Sciences; Brno, Czech Republic; 6 Department of Natural Drugs, Faculty of Pharmacy, University of Veterinary and Pharmaceutical Sciences; Brno, Czech Republic; 7 International Clinical Research Center, Brno University of Technology, Brno, Czech Republic

**Keywords:** haloperidol, guinea pig, oxidative stress, superoxide dismutase, glutathione reductase, glutathione-*S*-transferase

## Abstract

Guinea pigs (*Cavia porcellus*) were treated with haloperidol (HP), and free radical (FR) and ferric reducing antioxidant power (FRAP) assays were used to determine oxidative stress levels. Furthermore, the superoxide dismutase (SOD), glutathione reductase (GR) and glutathione-*S*-transferase (GST) activity levels were detected and glucose levels and the reduced and oxidized glutathione (GSH/GSSG) ratio were measured in HP-treated and untreated guinea pigs. The present study demonstrated that the administration of HP causes significant oxidative stress in guinea pigs (P=0.022). In animals treated with HP, the activity of GST was significantly increased compared with a placebo (P= 0.007). The elevation of SOD and GR activity levels and increase in the levels of glutathione (GSH) in HP-treated animals were not statistically significant. In the HP-untreated animals, a significant positive correlation was observed between oxidative stress detected by the FR method and GST (r=0.88, P=0.008) and SOD (r=0.86, P= 0.01) activity levels, respectively. A significant negative correlation between the levels of plasma glucose and oxidative stress detected by the FRAP method was observed (r=−0.78, P=0.04). Notably, no significant correlations were observed in the treated animals. In the HP-treated group, two subgroups of animals were identified according to their responses to oxidative stress. The group with higher levels of plasma HP had higher enzyme activity and reactive oxygen species production compared with the group with lower plasma levels of HP. The greatest difference in activity (U/μl) between the two groups of animals was for GR.

## Introduction

Haloperidol (HP) is a typical incisive antipsychotic drug. Chemically, it belongs to the butyrophenone series of antipsychotic compounds. Due to its marked central antidopaminergic action, HP is classified as a highly potent neuroleptic agent (1). It is prescribed in several diagnoses, such as psychosis, manic phases, hyperactivity, aggressiveness and acute delirium, and in certain cases, it is employed in long-term treatment. However, the use of typical neuroleptic drugs is limited by their side-effects and toxicity (2–6). Despite significant advantages provided by these remedies, patients using these drugs have to cope with the residual symptomatology which interferes significantly with their social and occupational life (7). Certain patients may develop disfiguring, disabling and potentially life-threatening adverse effects, including parkinsonian symptoms, tardive dyskinesia and neuroleptic malignant syndrome (8,9), whereas others are completely resistant to the treatment.

**Figure 1.**
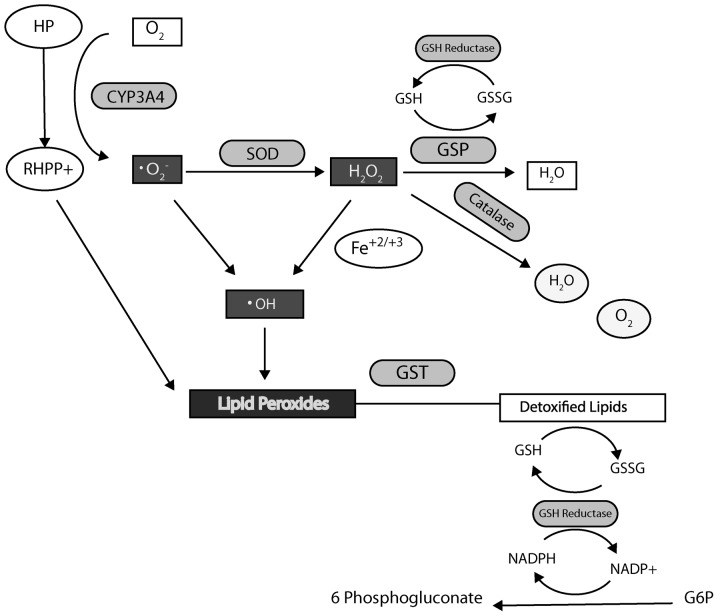
Antioxidant defense against HP toxicity. HP is oxidized to a toxic pyridinium metabolite (RHPP^+^) in the blood and brain by the specific iso-enzyme CYP 3A4. Superoxide may be produced by this metabolic pathway. RHPP^+^ causes lipid peroxidation which is removed by GST activity. GSH is oxidized to GSSG by this process. SOD is an enzyme that catalyzes the dismutation of superoxide into hydrogen peroxide. Hydrogen peroxide is removed by glutathione peroxidase and catalase. HP, haloperidol; GST, glutathione-*S*-transferase; GSH, reduced glutathione; GSSG, oxidized glutathionine; SOD, superoxide dismutase.

HP may have a direct cytotoxic effect via the production of toxic metabolites (10,11). Forsman *et al* reported the presence of reduced HP (RHP) as a major metabolite in the plasma of patients (12). The formation of these compounds is NADPH-dependent (12). Reduced HP is oxidized to a toxic pyridinium metabolite (RHPP^+^) by the specific isozymes CYP 450 and CYP 3A4 (13,14). HP is metabolically reduced in humans, but not in rats and the majority of other experimental animals, with the exception of guinea pigs (*Cavia porcellus*) (15). Thus, the molecular mechanisms of HP reduction may be studied using guinea pigs as a model for human HP metabolism. The main pathways of reactive oxygen species (ROS) production by HP treatment and the antioxidant system of defense are presented in Fig. 1.

Burkhardt *et al* observed that neuroleptics are able to inhibit NADH/ubiquinone oxidoreductase (complex-I) through their metabolites such as RHPP^+^(16). Complex-I inhibition is associated with the excessive generation of ROS. Chronic treatment with HP is known to induce oxidative stress due to the increased turnover of dopamine (17). Behl *et al* demonstrated that amyloid beta resistant cells were resistant to HP toxicity (18). This suggests a role for free radicals in HP-induced cell damage. Moreover, lipid peroxidation has been implicated to be a causal factor in the development of tardive dyskinesia and other movement disorders (19). Other evidence supporting this hypothesis includes elevated levels of lipid peroxidation in HP-treated rats (17), as well as in psychotic patients (20).

It appears to be clear that ROS are crucial in the generation of adverse HP side-effects. However, it is not known which of the main antioxidant enzymes have the greatest activity in the removal of HP-induced ROS. Thus, the determination of the oxidative stress and ROS-associated enzymes in an animal model was the aim of the present study. The level of oxidative stress was measured and compared in the plasma of 17 guinea pigs (10 HP-treated and 7 untreated). Furthermore, the superoxide dismutase (SOD), glutathione reductase (GR) and glutathione-*S*-transferase (GST) activity detection was recorded, as well as the glucose levels and reduced and oxidized glutathione (GSH/GSSG) ratio.

## Materials and methods

### Animals

All animal experiments in the present study were performed inaccordance with the recommendations of the European Community Guide for the Care and Use of Laboratory Animals and followed the guidelines for animal treatment approved by local authorities.

Four-month-old guinea pigs were obtained from Velaz (Prague, Czech Republic) and during the study they were kept in the Animal Facility of Masaryk University (Brno, Czech Republic). A total of 10 animals were treated with HP and 7 with a physiological solution (saline) applied intraperitoneally for 21 successive days. The total dose of HP was 4,200 μg per 100 g of body mass.

### Determination of low-molecular-mass thiols and HP

The high performance liquid chromatography with electrochemical detection (HPLC-ED) system consisted of two solvent delivery pumps operating in the range of 0.001–9.999 ml/min (Model 582; ESA Inc., Chelmsford, MA, USA), Zorbax Eclipse AAA Column (4.6x150 mm 3.5-micron particle size; Varian Inc., Paulo Alto, CA, USA) and a CoulArray electrochemical detector (Model 5600A, ESA Inc.). The sample (30 μl) was injected using an autosampler (Model 542; ESA Inc.). The HPLC-ED experimental conditions were as follows: the compositions of the mobile phases were 80 mM trifluoroacetic acid (A) and methanol (B). The mobile phases were mixed in a gradient from 3% B in the 1st min, 10% B between the 2nd and 6th minute and 98% B from the 7th minute of the separation. The flow of the mobile phase was 0.8 ml/min, the temperature of the separation was 40°C, the working electrode potential was 900 mV, the detector temperature was 30°C and each measurement was performed in triplicate. The signals of GSH, GSSG and HP were quantified as the sum of the current responses from all working electrodes. For the real sample measurements, the shift of the retention time was of ∼±2%.

### Spectrometric measurement

Spectrophotometric measurements were carried out using an automated chemical analyzer BS-400 (Mindray, Shenzhen, China). The analyzer was composed of a cuvette space (37±1°C), reagent space with a carousel for reagents (4±1°C), sample space with a carousel for the preparation of samples and an optical detector. The transfer of samples and reagents was performed by a robotic arm equipped with a dosing needle (dosage error ≤5% of volume). The cuvette contents were mixed by an automatic mixer, including a stirrer, immediately after the addition of reagents or samples. Contamination was reduced by a rinsing system which included rinsing of the dosing needle and stirrer with MilliQ water. For detection, the following wavelengths were usable: 340, 380, 412, 450, 505, 546, 570, 605, 660, 700, 740 and 800 nm.

### Determination of SOD

Kit 19160 SOD (Sigma Aldrich, St. Louis, MO, USA) was used for the assay of SOD (EC 1.15.1.1.). First, 200 μl R1 reagent (WTS solution diluted 20-fold with buffer) was pipetted into a plastic cuvette and incubated at 37°C for 108 sec. Subsequently, 20 μl of sample was added and in 378 sec, the reaction was started by adding 20 μl R2 reagent (enzyme solution diluted 167-fold with buffer). The reaction was incubated for 72 sec and then absorbance was measured at λ=450 nm. The kinetic reaction was measured for 108 sec and the absorbance was recorded every 9 sec.

### Determination of GR

A GR Assay Kit (Sigma Aldrich) was used for the GR activity determination. Reagents R1 and R2 were prepared by dissolving in assay buffer (100 mM potassium phosphate buffer, pH 7.5, with 1 mM EDTA). The R1 reagent (260 μl; 1.15 mM oxidized GSH in the assay buffer) was added with 10 μl of sample and 30 μl R2 reagent (1 mM NADPH in GR assay buffer) into a plastic cuvette. The decrease in absorbance was measured at 340 nm using a kinetic program for 1,260 sec.

### Determination of GST

The method used was based on the GST-catalyzed reaction between GSH and the GST substrate, 1-chloro-2,4-dinitrobenzene (CDNB). GST substrate has the broadest range of isozyme detectability (e.g., α, μ, π and other GST isoforms). Under certain conditions, the interaction between GSH and CDNB is dependent on the presence of active GST. The GST-catalyzed formation of GS-DNB produces a dinitrophenylthioether which may be detected spectrophotometrically at 340 nm. A 180-μl volume of reactants consisting of 2 mM CDNB and PBS (1.4 mM NaH_2_PO_4_ and 4.3 mM Na_2_HPO_4_, pH 7.4; 1:19, v/v, 37°C) was added to the sample in a plastic microtube. Furthermore, 12.5 mM GSH (30 μl) in 0.1 M phosphate buffer (pH 7.4) was added. A wavelength of 340 nm was used to determine the GST activity.

### Determination of antioxidant activity by the ferric reducing antioxidant power (FRAP) method

The FRAP method is based on the reduction of complexes of 2,4,6-tripyridyl-*s*-triazine (TPTZ) with ferric chloride hexahydrate (FeCl_3_·6H_2_O); these substances are almost colorless and eventually slightly brown. Following the reduction, blue ferrous complexes are formed. The reagents were prepared as follows: solution 1 contained 10 mmol/l TPTZ in 40 mmol/l hydrochloric acid. Solution 2 contained 20 mmol/l ferric chloride hexahydrate in ACS water. Solution 3 contained 20 mmol/l acetate buffer, pH 3.6. These three solutions (TPTZ, FeCl_3_ and acetate buffer) are mixed in a 1:1:10 ratio.

The procedure for the determination was taken from the study by Sochor *et al*(21). After 150 μl of reagent was injected into a plastic cuvette with the subsequent addition of 3 μl sample, the absorbance was measured at 605 nm for 12 min. The difference between absorbance at the last (12th) and the 2nd minute of the assay procedure was used to calculate the antioxidant activity.

### Determination of antioxidant activity by the free radicals (FR) method

This method is based on ability of chlorophyllin (the sodium-copper salt of chlorophyl) to accept and donate electrons with a stable change of maximum absorption. This effect requires an alkaline environment and the addition of a catalyst.

The procedure for the determination was taken from the study by Sochor *et al*(21). Reagent (150 μl) was injected into a plastic cuvette with the subsequent addition of a 6 μl sample. The absorbance was measured at 450 nm in the second and last (12th) minute of the assay. The difference between two absorbances was considered to be the output value.

### Determination of glucose

First, 200 μl of the reagent (0.1 M phosphate buffer, pH 7.5, 0.75 mM phenol, 0.25 mM 4-amino-antipyrine (4-AAP), glucose oxidase ≥15 kU/l, peroxidase ≥1.5 U/l) was pipetted into a plastic cuvette with 20 μl of the sample. The absorbance was then measured for 10 min at λ=505 nm. To calculate the absorbance, the values of the sample, reagents and reaction mixture after 10 min of incubation with the sample were used.

### Statistical analysis

Software Statistica 10 (StatSoft Inc., Tulsa, OK, USA) was used for the statistical analysis. The Shapiro-Wilk test was used to assess normality. Mann-Whitney U tests were used to evaluate the differences between the groups. Simple linear correlations were performed to reveal the associations between the variables. Tree clustering was used to visualize the distribution of variables and K-means clustering was used to divide the cases into clusters. Unless noted otherwise, P<0.05 was considered to indicate statistically significant differences.

## Results

**Figure 2.**
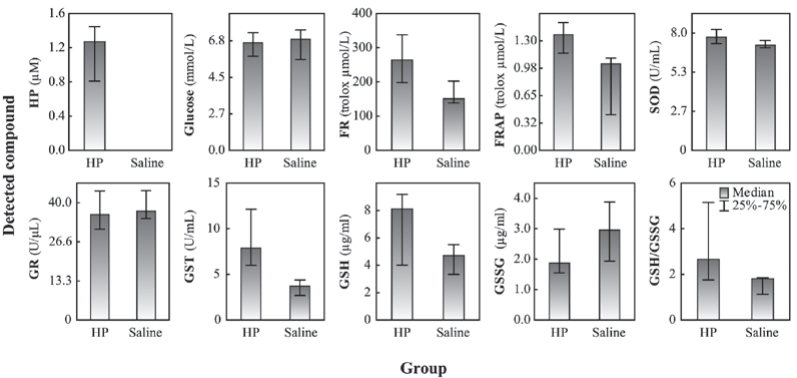
Levels of oxidative stress and enzyme activities in blood plasma of guinea pigs. HP, haloperidol; saline, solution of 0.90% w/v of NaCl; FR, free radicals method was used for the determination of oxidative stress; FRAP, ferric reducing antioxidant power method was used for the determination of oxidative stress; SOD, superoxide dismutase; Glc, glucose; GR, glutathione reductase; GST, glutathione-*S*-transferase; GSH, glutathione; GSSG, oxidized glutathione; GSH/GSSG, ratio between reduced and oxidized glutathione.

A total of 10 guinea pigs were treated with HP and seven with saline. The oxidative stress and enzyme activity levels in plasma were measured. The SOD, GR and GST activity levels were detected and the glucose levels and the GSH/GSSG ratio were measured. HP was present in the plasma of treated animals, while it was undetectable in the untreated animals. Animals treated with HP exhibited significantly increased activity of GST (P= 0.007). The elevation of SOD and GR activity levels and an elevated level of GSH in HP-treated animals were observed but not significant. Also, the GSH/GSSG ratio was not shifted due to the oxidative state and no significant differences were observed in the glucose levels between the control and HP-treated animals (Fig. 2). The present study demonstrates that the administration of HP causes significant oxidative stress, measurable by spectrometric FR and FRAP assays (P=0.02 and P=0.05, respectively).

**Figure 3.**
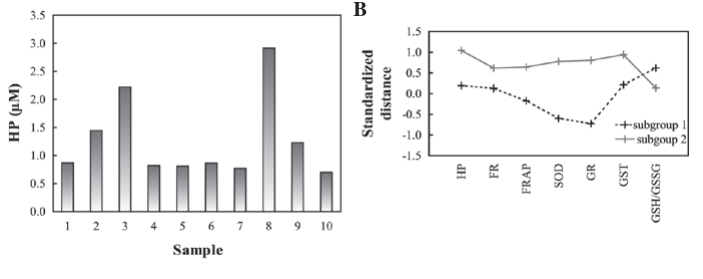
(A) Plasma levels of HP in individual animals of the treated group. (B) Animals were divided into two subgroups by HP and ROS levels using K-means cluster analysis. The levels of ROS and HP within these subgroups are shown. Concentrations were recalculated to a standardized distance to allow comparison. HP, haloperidol; FR, free radicals method; FRAP, ferric reducing antioxidant power method; SOD, superoxide dismutase; GR, glutathione reductase; GST, glutathione-*S*-transferase; GSH/GSSG, ratio between reduced and oxidized glutathione.

The plasma levels of HP in the treated animals varied considerably (range, 0.7–2.9 μM), although all animals received the same dose of HP according to their body mass (Fig. 3A). Using K-means clustering, the HP-treated guinea pigs were divided into two clusters according to levels of HP, oxidative stress and ROS-enzymes (Fig. 3B and Table I). Two characteristic contrasting subgroups of animals were observed: the first subgroup had higher HP, FR, FRAP, SOD, GR and GST values and lower GSH/GSSG ratios, while the second had lower HP, FR, FRAP, SOD, GR and GST values and higher GSH/GSSG ratios.

**Figure 4.**
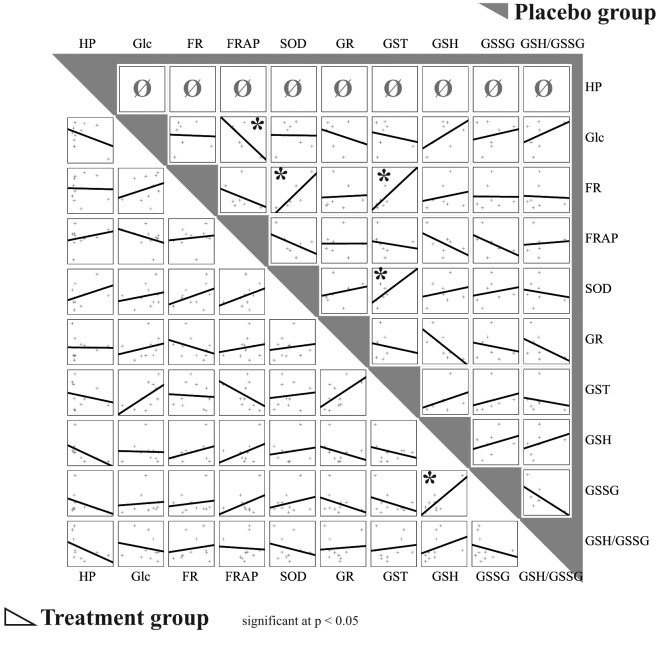
Correlations between observed parameters in treated and placebo (saline) groups. Treatment group bottom left, placebo group top right (gray). ^*^P<0.05. HP, haloperidol; FR, oxidative stress measured by free radicals method; FRAP, oxidative stress measured by ferric reducing antioxidant power method; SOD, superoxide dismutase; Glc, glucose; GR, glutathione reductase; GST, glutathione-*S*-transferase; GSH, glutathione; GSSG, oxidized glutathione; GSH/GSSG, ratio between reduced and oxidized glutathione.

The greatest difference in activity (U/μl) between the two groups of animals was observed for GR. In the placebo group, significantly positive correlations were observed between oxidative stress detected by the FR method (r=0.88, P=0.008) and GST activity, as well as between oxidative stress detected by the FR method (r= 0.86, P= 0.01) and SOD activity. A significant negative correlation was observed between the level of plasma glucose and oxidative stress detected by the FRAP method (r=−0.78, P=0.04). There was also positive correlation between SOD and GST activity (r=0.80, P=0.03) in the plasma of untreated animals. By contrast, no similar significant correlations were observed in the HP-treated animals (Fig. 4).

## Discussion

Adult guinea pigs were used to study the molecular mechanisms of HP metabolism. Since the observation of the initial activity of the antioxidant enzymes was being investigated, the animals were treated for a relatively short period (3 weeks). According to Lawler *et al*, increased levels of the antioxidant enzymes may be detected immediately after ROS production (22). Cells respond to acute oxidative stress by the induction of the expression of genes products which protect the cell. However, chronic oxidative stress causing long-term increased production of these enzymes is extremely burdensome for the cell. As a result, although ROS exposure remains present, the production of enzymes gradually decreases (23).

Although all the experimental animals were treated with a total dose of 4,200 μg of HP per 100 g of body mass, the levels of HP in their plasma varied considerably. This may indicate a high interindividual variability in the activity of the enzymes involved in the metabolism of HP in guinea pigs.

When the activity of the antioxidant enzymes was compared between the treated and placebo groups, only one statistically significant difference was identified. In animals treated with HP, significantly increased activity of GST was observed. GSTs are evolutionarily conserved enzymes important in the detoxification of numerous xenobiotic compounds. These enzymes catalyze the conjugation of GSH to electrophilic substrates, thus producing compounds that are generally less reactive and more soluble. This facilitates the removal of these compounds from the cell via membrane-based GSH conjugate pumps. The broad substrate specificity of GSTs allows them to protect cells against a wide range of toxic chemicals (24). The GSH peroxidase activity of a number of GST proteins also suggests that they may be important in organic peroxide detoxification (25). GSTs are able to conjugate GSH to these toxic reactive compounds, forming 4-hydroxynonenal and cholesterol α-oxide which are generated during the oxidation of membranes (26). GSTs may have a wider role in the response to cellular stress beyond their enzymatic activity. In particular, GSTs have been shown to act as stress-sensitive inhibitors of the mammalian stress-activated protein kinase c-Jun NH_2_-terminal kinase. This helps to maintain c-Jun NH_2_-terminal kinase in an inactive form in unstressed cells (27). Based on the increased activity of GST and in accordance with the studies of Shivakumar and Ravindranath and Pai *et al*(17,20), peroxidation of membrane lipids was proposed to be the main mechanism of HP adverse effects. This hypothesis is be further supported by the observation that HP tends to decrease the permeability of a number of biological membranes to various inorganic and organic molecules, including water, and that it exerts this effect at minute concentrations (28).

The present study demonstrates that the administration of HP causes significant oxidative stress which is measurable by spectrometric FR and FRAP assays but not by the GSH/GSSG ratio. This ratio was not changed relative to the oxidative state. It may indicate that 3 weeks of HP treatment are not long enough to deplete the GSH supplies of healthy guinea pigs. This is in agreement with Pai *et al* who demonstrated no changes in GSH levels after the first two weeks of HP administration in psychotic patients (20).

In the placebo group, significant positive correlations were observed between oxidative stress detected by the FR method and GST and SOD activity levels, respectively, which is in compliance with activation of antioxidant enzymes by oxidative stress (22). This correlation was observed in the untreated group only, although oxidative stress was significantly higher in the treated group. A significant negative correlation was observed between the level of plasma glucose and oxidative stress detected by the FRAP method, but only in the placebo group. In the treated group, no significant correlations were observed. It appears that the mechanisms of defense against small, relatively-stable daily oxidative stress are different from those activated by acute high stress.

Two groups of animals were identified according to how they responded to oxidative stress (high plasma HP and high oxidative parameters group and low plasma HP and low oxidative parameters group). This appeared to be the reason for the lack of significance of the correlations between oxidative stress detected by the FR method and GST and SOD activity levels, respectively, in the treated animals. However, these two sub-groups were too small to conduct the same further statistical assessments as for all the groups together. These results demonstrate the great variability in the activation of antioxidant enzymes by HP detoxification in guinea pigs.

## Figures and Tables

**Table I.** Concentrations of HP, oxidative stress parameters and oxidative stress enzymes in the placebo and treatment groups and two subgroups (clusters) of treated animals (shown as mean ± 1 SD).

Group	Number	HP (μM)	Glc (mM)	FR (trolox μM)	FRAP (trolox μM)	SOD (U/l)	GR (U/l)	GST (U/l)	GSH/GSSG
Placebo	7	0±0	6.70±1.03	194±42	1.17±1.07	7.94±0.38	35.0±5.66	4.90±2.38	1.8±1.0
Treatment	10	1.26±0.74	6.13±0.94	287±81	1.36±1.49	7.97±0.85	32.7±8.99	9.33±3.16	3.4±2.2
Subgroup 1	4	0.90±0.21	6.42±0.92	272±94	0.71±1.34	7.80±0.50	29.0±8.76	8.89±2.99	4.0±2.5
Subroup 2	5	1.62±0.93	5.83±1.07	276±70	1.68±1.24	7.78±0.86	36.8±9.04	10.53±3.02	3.1±2.0

HP, haloperidol; Glc, glucose; FR, oxidative stress measured by free radicals method; FRAP, oxidative stress measured by ferric reducing antioxidant power method; SOD, superoxide dismutase; GR, glutathione reductase; GST, glutathione-*S*-transferase; GSH, glutathione; GSSG, oxidized glutathione; GSH/GSSG, ratio between reduced and oxidized glutathione.
